# Nanotechnology-Based Therapies for Preventing Post-Surgical Adhesions

**DOI:** 10.3390/pharmaceutics17030389

**Published:** 2025-03-19

**Authors:** Zi Yi Teo, Samyuktha Dhanalakshmi Senthilkumar, Dinesh Kumar Srinivasan

**Affiliations:** 1 Yong Loo Lin School of Medicine, National University of Singapore, Singapore 117597, Singapore; e0974441@u.nus.edu (Z.Y.T.); e1032057@u.nus.edu (S.D.S.); 2 Department of Anatomy, Yong Loo Lin School of Medicine, National University of Singapore, Singapore 117594, Singapore

**Keywords:** adhesion, post-operation, nanotherapeutics, nanocomposites, hydrogels, nanofibers

## Abstract

Adhesions are the body’s natural response to various inflammatory causes, with surgery being the most common cause. However, the formation of postoperative adhesions can lead to significant complications, including intestinal obstruction and chronic pain. To prevent such postoperative complications associated with adhesions, developing effective strategies for adhesion prevention has been a major focus of research. Currently, several therapeutic models have been developed to achieve this objective. These include pharmaceuticals, inert polymers, functional biomaterials, and nanotherapeutics. Among the various strategies developed, nanotherapeutics, though still in its early stages, has shown promise as a potential approach. Other therapeutic models are associated with adverse side effects and complications related to their application. On the other hand, nanotherapeutic models are able to overcome the limitations of the other strategies and provide their own set of unique advantages. Hence, nanotherapeutics represents a promising area for further research. Further efforts should be made to refine existing nanotherapeutics for clinical application while also addressing associated safety and ethical concerns related to their use in medical practice. Therefore, this article aims to review the various nanotherapeutic approaches developed for the prevention of postoperative adhesions, explore their regulatory pathways, and discuss associated safety and ethical concerns.

## 1. Introduction

**Figure 1:**
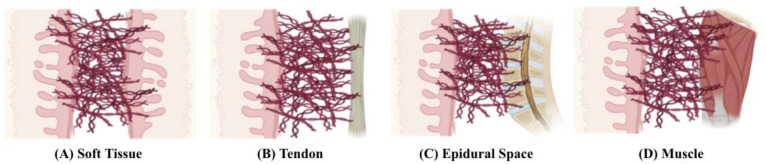
Adhesions form most commonly between planes of soft tissue (**A**). They can also form between soft tissue and tendon (**B**), epidural space (**C**), and muscle (**D**). Adapted from Park et al. 2020, distributed under the terms and conditions of the Creative Commons Attribution (CC BY) licence (http://creativecommons.org/licenses/by/4.0/) (accessed on 16 December 2024) [1].

Adhesions are fibrous bands that form between different layers of the body, usually occurring between soft tissue and other tissue types [1], as shown in Figure 1. Adhesions are mostly found in the abdomen and pelvis as a natural response to various inflammatory causes, with surgery being the most frequent cause [2]. Given that the organs in the abdominopelvic cavity are all lined by the peritoneum, the pathophysiology of postoperative adhesion formation in the abdomen and pelvis is similar.

Although adhesion formation plays a vital role in the post-surgical healing process, it is also known to cause significant complications. For example, the formation of peritoneal adhesions inhibits the movement of organs, which can then cause bowel obstruction as well as chronic abdominal pain [3]. Consequently, various methods have been developed to prevent postoperative adhesions. While mechanical barriers are the most employed approach, their use is often limited to cases involving small incisions and restricted surgical access. Furthermore, mechanical barriers can be challenging to apply to tissues with complex geometries and may adhere aggressively to the surgeon’s gloves during use [4].

With the recent advancements in nanotechnology, there has been a growing interest in its potential to prevent postoperative abdominal adhesions. However, research and clinical experience in this area remain limited. Thus, this paper aims to review the various nanotherapeutic approaches which prevent postoperative abdominal adhesions and their potential for future clinical applications.

### Pathophysiology of Postoperative Abdominal Adhesion Formation

**Figure 2:**
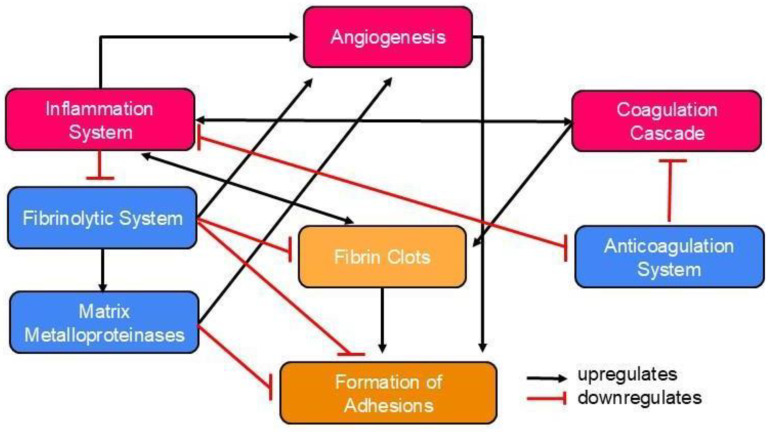
There are many pathways which interact with each other during the process of adhesion formation. Fibrin deposition, which leads to the production of fibrin clots, is mainly regulated by the coagulation cascade, inflammation system, anticoagulation system, and fibrinolytic system. Other elements which influence adhesion formation include matrix metalloproteinases and angiogenesis. When fibrin deposition exceeds fibrinolysis, adhesions are formed.

Postoperative adhesions occur in 50–90% of all open abdominal surgeries. They represent a significant clinical problem that affects hundreds of millions of patients each year [5]. These adhesions can lead to serious complications, making adhesion prevention a critical area of focus. Within ten years of abdominal or pelvic surgery, 35% of patients experienced readmission an average of 2.1 times due to disorders directly or potentially related to adhesions [6]. To develop successful preventive strategies, understanding how various cells and factors contribute to adhesion formation is necessary. In simple terms, adhesions occur when fibrin deposition outpaces fibrinolysis. However, the process of postoperative adhesion formation is complex, involving various systems, pathways, and cellular interactions [7]. The interactions between the main pathways and the cell types involved are illustrated in Figure 2.

Surgery disrupts the epithelium or mesothelium on the basement membrane. The subsequent exposure of the basement membrane triggers the infiltration of neutrophils and monocytes, which stimulates inflammation and the secretion of fibrin-rich exudate during the initial healing phase [8]. Activated platelets release cytokines, chemokines, fibrinogen, and fibrin which recruit macrophages, neutrophils, and epithelial cells to the injury site, supporting inflammation and the healing process [9]. At the same time, platelet aggregation and coagulation occur to minimise blood loss. The activated pro-coagulation factors culminate in fibrin monomer formation, in a process mediated by thrombin. The fibrin monomers will then aggregate with the activated platelets to form fibrin clots [10]. Inflammation and coagulation are tightly interconnected. Inflammatory cytokines regulate the coagulation cascade, while coagulation proteases regulate inflammation by influencing cytokine and growth factor production [11].

Haemostasis requires the tight regulation of coagulation through the anticoagulation system. In particular, the protein C pathway influences the formation of thrombin when thrombin binds to thrombomodulin. This upregulates the activation of protein C, and inhibits fibrin formation as well as coagulation [12]. In addition, inflammation downregulates the protein C pathway and vice versa [11]. The fibrinolytic system involves plasminogen activators (PA) and plasminogen activator inhibitors (PAI). The components of the fibrinolytic system rely on an intact mesothelium, and they play a vital role in fibrin degradation and limiting adhesion formation [9]. The fibrinolytic system promotes the action of matrix metalloproteinases, which degrade extracellular matrix (ECM) components [13].

Angiogenesis involves ECM degradation and neovascularization [14]. Although the exact mechanism remains unclear, it is believed to enhance the formation of adhesions directly recruiting pericytes, which then adopt a fibroblastic phenotype [15]. Coagulation and inflammation promote fibrin deposition, while anticoagulation and fibrinolysis reduce deposition and break down the matrix [7]. Persistent fibrin clots allow for the attachment of fibroblasts and inflammatory cells. Along with vascular formation, this promotes an organised matrix deposition, leading to adhesion formation. When the integrity of the mesothelium and basal membrane is compromised, fibrin deposition exceeds fibrinolysis, causing adhesions [7].

In peritoneal adhesion formation, fibroblasts transform into an adhesion phenotype under hypoxia [16], upregulating vascular endothelial growth factor (VEGF) production to reoxygenate hypoxic tissue, which is represented by fibrin clots [17]. The degradation of ECM also upregulates adhesion formation [9].

## 2. Therapeutic Models to Prevent Postoperative Abdominal Adhesions

Bowel obstruction is a common complication of postoperative adhesions. In some cases, surgical adhesiolysis is required, in which adhesion bands are dissected to relieve the obstruction. However, adhesiolysis is expensive and time-consuming, can lead to longer hospitalisations, and can negatively impact patients’ quality of life. Thus, there is greater interest in methods to prevent adhesion formation [2].

### 2.1. Surgical Techniques

There are several intraoperative measures to prevent postoperative adhesions. These measures include gentle tissue handling, ensuring thorough hemostasis, and adding more than 5% nitrous oxide to the carbon dioxide pneumoperitoneum during laparoscopic surgeries [18].

Laparoscopic surgeries result in fewer and less severe post-surgical adhesions compared to open surgeries. In a study of 151 patients, 78.9% of those who underwent open colorectal resection developed adhesions, compared to 37.7% of those who had laparoscopic resection [19]. Another study reported a 1.7% readmission rate for adhesion-related issues among laparoscopic surgery patients, compared to a higher rate of 4.3% among open surgery patients [20]. However, since laparoscopic techniques reduce but do not eliminate adhesions, it is essential to develop additional strategies to prevent their formation.

### 2.2. Pharmaceutical Strategies

Adjuvants are agents that either disrupt adhesion formation pathways or enhance inhibitory pathways. Early studies on fibrin in adhesions explored fibrinolytic agents like fibrinolysin, pepsin, trypsin, and PA [21]. These agents promote fibrinolysis or directly target fibrin clots. Current studies examine tissue plasminogen activator (t-PA), plasminogen activator inhibitor-1 (PAI-1), and streptokinase. However, their effectiveness is limited, and side effects like bleeding have been observed.

Another approach is the regulation of local inflammation. While hyaluronic acid (HA) is primarily considered to be a mechanical barrier, it dissolves fibrin and is anti-inflammatory in nature [22]. Although HA is an ideal anti-adhesion material, its rapid resorption limits its ability to prevent adhesions. Hence, methods to prolong and maximise its anti-adhesive properties are being studied. Other drugs that have been studied include resveratrol [23] and pirfenidone [24], which target inflammatory cytokines, such as transforming growth factor-beta and tumour necrosis factor-alpha (TNF-α). Although these drugs were promising, they have not been clinically evaluated and may lead to undesirable side effects [25]. Other agents, such as anticoagulants and antioxidants [26], have also been studied. Although some have shown promise in animal models, there are no data supporting their efficacy in humans.

Therapies that integrate mechanical barriers and drugs may be a feasible approach to preventing adhesions. However, some combinations such as Interceed^®^ with heparin, and Seprafilm^®^ with vitamin E, have shown no improvement in efficacy [27,28]. Others have shown only minimal improvements in animal models [29]. Although the use of adjuvants holds promise in theory, they present inherent limitations. Due to the complexity and interconnectedness of these pathways, studies suggest that a single extracellular mediator is insufficient to prevent adhesions. Instead, the use of various synergistic agents may be necessary [30,31]. Further challenges include the ability to deliver agents in a localised fashion, and the permanency and side effects of the agents [29,32]. Therefore, further research and clinical trials are needed to assess the safety and efficiency of these agents in various surgical procedures [7].

### 2.3. Mechanical Barriers

**Figure 3:**
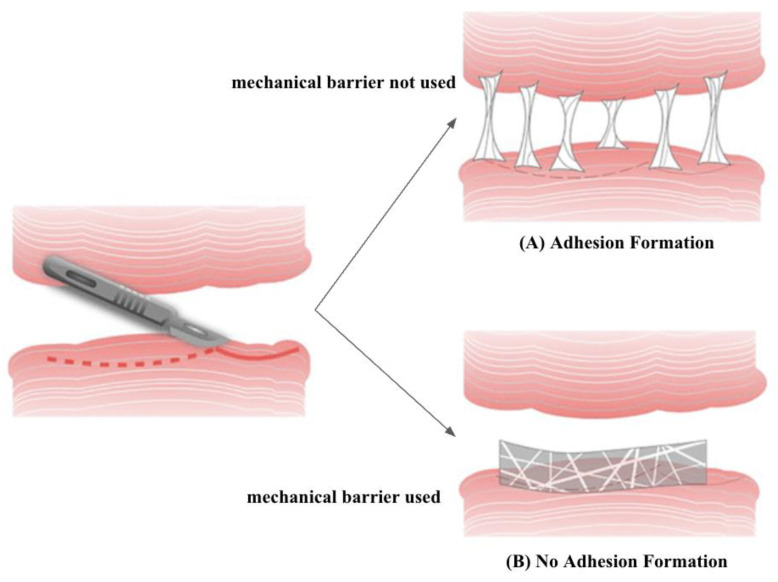
(**A**) When a mechanical barrier is not used, postoperative adhesions will form at the surgical site. (**B**) The use of a mechanical barrier at the surgical site will help to prevent the formation of postoperative adhesions. Adapted from Klicova et al. 2023, distributed under the terms and conditions of the Creative Commons Attribution (CC BY) licence (https://creativecommons.org/licenses/by-nc-nd/4.0/) (accessed on 16 December 2024) [33].

Apart from pharmaceutical strategies, efforts have also been made to develop mechanical barriers, including solid polymers, gels, and liquids, which have been widely used in various tissues to prevent adhesions [33] (Figure 3). However, in laparoscopic surgeries, liquid and gel barriers are more convenient to apply than solid ones [34]. Significant research has explored natural and synthetic polymers as barriers in the in vivo and clinical context [22]. In addition, combining mechanical barriers of different structures offers various possibilities that are being investigated [35]. Recently, tissue grafts, including allogeneic amniotic membranes, have been studied, but they yielded poor results in peritoneal adhesion prevention [7].

Recently, a randomised clinical trial involving 127 patients undergoing uterine myomectomy showed that Seprafilm^®^ effectively reduced adhesions [36]. Similarly, Interceed^®^ reduced the formation of adhesions from 76% to 41% in a randomised multicentre clinical study involving 63 patients undergoing bilateral pelvic sidewall adhesiolysis [37]. The safety and anti-adhesive potential of Adept^®^ was demonstrated in a randomised double blind clinical study involving 402 patients undergoing laparoscopic gynaecological surgery [38]. Sprayshield™ demonstrated a reduced incidence and severity of adhesion formation in a randomised single blind study which involved 11 patients diagnosed with ulcerative colitis and familial adenomatous polyposis [39]. In a trial consisting of 52 patients, Hyalobarrier^®^ was found to be safe to use and demonstrated anti-adhesive properties in patients undergoing laparoscopic gynaecological surgery [40]. Table 1 presents a comprehensive overview of several of the key studies that highlight the use of mechanical barriers.

Overall, although mechanical barriers in the form of films are commonly used, their application to irregular surfaces and cavities is challenging. This is due to their fragility, difficulty in handling, incompatibility with minimally invasive laparoscopic or catheter-based procedures, and limited efficacy (~25%). Novel technologies are needed to overcome clinical limitations [41].

### 2.4. Gene Therapy

With the recent advances in molecular biology, gene therapy is a feasible substitute or complementing approach to adhesion prevention. Some strategies include delivering t-PA genes via viral vectors to promote fibrinolysis, using small interfering ribonucleic acid to reduce hypoxic gene expression, and decreasing the action of fibrinolysis inhibitors [26]. However, these methods have yielded modest results. Similarly, transferring the hepatocyte growth factor gene via viral vectors demonstrated only some reduction in peritoneal adhesions in animal models [42].

## 3. Nanotherapeutics for the Prevention of Abdominal Adhesions

Many innovative nanotheranostic systems enable targeted drug delivery in various biomedical fields. In preventing abdominal adhesions, the available nanotherapeutic solutions include nanocomposites, hydrogels and nanofibers.

### 3.1. Nanocomposites

Nanocomposites offer exceptional advantages in preventing postoperative adhesions, including their biocompatibility and enhanced mechanical characteristics. Furthermore, a wide range of nanosized organic and inorganic substances can be combined to generate diverse nanocomposites with distinct physicochemical, physical, and biological properties [43]. Wei et al. (2022) observed that poly(dopamine) human keratinocyte growth factor (PDA-KGF) nanoparticles, when combined with HA, were effective in preventing postoperative adhesions in animal models. This was achieved by reducing collagen deposition, fibrosis and inflammatory responses, while also promoting mesothelial cell repair in the injured peritoneum. The PDA-KGF nanoparticles had a globular morphology, an average diameter of about 200 nm, and demonstrated a slow release of KGF. This slow release helped prolong the bioavailability of KGF, which would otherwise be short-lived [44]. Thus, the incorporation of KGF into the nanoparticles maximised the previously established effects of KGF in promoting mesothelial cell regeneration and enhancing fibrinolytic activity [45]. Hence, KGF plays a key role in the prevention of postoperative adhesions.

A biologically targeted, photo-crosslinkable nanopatch (pCNP) has been developed for postoperative adhesion prevention. It consists of two nanoparticles: nanoparticle A and nanoparticle B. Nanoparticle A has an average hydrodynamic diameter of 166.1 ± 1.8 nm and a negatively charged surface of −12.1 ± 0.3 mV. Nanoparticle B has an average hydrodynamic diameter of 175.1 ± 28.7 nm and a positively charged surface of 23.0 ± 3.0 mV. Nanoparticle A will bind to the site of injury and deliver dexamethasone 21-palmitate (DXP), an anti-inflammatory drug that prevents adhesion formation. Based on the study’s trials, which were conducted in a rat parietal peritoneum excision model, the pCNP developed was more effective in preventing surgical adhesions than Seprafilm^®^. Furthermore, it did not show any significant toxicity. The effectiveness of the pCNP in preventing adhesions can be attributed to two characteristics. Firstly, its nanoparticles specifically target the site of injury through peptides that bind to collagen on the exposed basement membrane. Secondly, the opposite charges of the two nanoparticles enable the formation of ionic interactions that give rise to a barrier with sufficient density at the site of injury. pCNP releases DXP and enhances the anti-adhesive effects of the nanoparticles due to its ability to reduce inflammation [46].

### 3.2. Hydrogels

Hydrogels are polymers with three-dimensional network structures that can absorb large amounts of fluid [47]. Their mechanical properties can be modified for use in various contexts [48]. As such, hydrogels can be designed to possess favourable characteristics that enable them to address the limitations of current anti-adhesive products. This makes hydrogels an appealing alternative to commercially available barriers.

A recent study developed a nanocomposite hydrogel composed of collagen aldehydeylated poly(ethylene glycol) cysteine human serum albumin-18His protein and docetaxel (Col-APG-Cys@HHD). This hydrogel is capable of preventing intraperitoneal adhesions while simultaneously inhibiting tumour growth. Composed of collagen dissolved in ultrapure water and then combined with APG, the hydrogel adheres to tissue on one side due to the collagen. The other side is coated with cysteine. The hydrogel delivers cysteine to the site of injury, which helps prevent the adhesion of proteins, blood cells, and fibroblasts. This aids in controlling fibrin deposition, the primary mechanism for reducing postoperative adhesion formation. The HSA-18His fusion protein had a molecular weight of around 70 kDa. The HSA-18His protein has a drug loading rate of 10% and thus could be loaded with about nine docetaxel molecules. It is self-organised into uniform, spherical HHD nanoparticles around 100 nm in diameter. Histidine’s sensitivity to acidic environments and its ease of protonation enhanced its solubility, promoting the disintegration of HHD nanoparticles and drug release. The design of Col-APG-Cys@HHD overcomes the limitations of traditional hydrogels, which lack a combination of tissue adhesion and anti-biological contamination. Furthermore, it provides a means for preventing both postoperative abdominal adhesions and tumour recurrence with minimal side effects [49].

Wang et al. (2019) designed a naproxen-loaded chitosan hydrogel that combined the anti-adhesion properties of chitosan hydrogels with the analgesic and anti-inflammatory properties of naproxen. The chitosan hydrogel was prepared by mixing chitosan powder with an aqueous acetic acid solution and β-glycerolphosphate disodium salt pentahydrate (β-GP). It primarily acts as a barrier and facilitates the delivery of naproxen nanoparticles, which are the key factors in preventing adhesions. The developed naproxen nanoparticles leverage the known anti-inflammatory properties of naproxen. However, the novel formulation allows for their injection alongside the chitosan hydrogel during minimally invasive surgeries. These characteristics enable the hydrogel to combat the formation of postoperative adhesions via the reduction in inflammation. The naproxen nanoparticles, with a 33 nm diameter and 0.16 polydispersity index, released 32% of naproxen in 24 h, and 68% over 6 days. Upon an evaluation of the hydrogel’s efficacy in preventing adhesions in a rodent abdominal adhesion model, it was found to be as effective as commercial products in preventing postoperative adhesions. It also demonstrated stable drug release behaviour [4].

A novel “nanoengineered hydrogel” barrier, which is mainly composed of silicate nanoplatelets and poly(ethylene oxide) (PEO), has been developed to prevent postoperative adhesions. This barrier leverages the knowledge that PEO can inhibit the infiltration and adhesion of fibroblasts, thereby controlling fibrin deposition and playing a crucial role in preventing postoperative adhesions. The disc-shaped silicate nanoplatelets (thickness ~0.92 nm, diameter ~25 nm) possess unique electrostatic properties, with negatively charged surfaces and positively charged edges. This leads to nanoscale surface-to-edge attraction and spontaneous superstructure formation. These characteristics enable self-assembly and gelation upon dissolution in water. The dual charges impart non-Newtonian and shear-thinning behaviour to the system, making it injectable and sprayable under stress, with immediate mechanical recovery. This advantageous feature allows for its application to large surface areas even in minimally invasive interventions, and enables it to adapt to complex anatomies by coating their surface. Furthermore, PEO, a biocompatible polymer with a molecular weight of 20,000, possesses unique antifouling properties, low immunogenicity, and minimal binding sites for cell adhesion or protein adsorption. As a result, PEO effectively prevents the infiltration of collagen-secreting cells and cellular growth. Hence, this nanoengineered hydrogel is effective in preventing postoperative adhesions in a variety of surgical procedures. Of the three hydrogel formulations studied, the one containing 10% by weight of silicate nanoplatelets, and 3% by weight of PEO, was the most effective at preventing postoperative adhesions. However, all of the hydrogel formulations studied showed better outcomes than Seprafilm^®^ [41].

### 3.3. Nanofibers

Planar nanofibrous layers prevent postoperative adhesions through two main mechanisms. The first is their ability to act as a mechanical barrier separating layers of soft tissue during the healing process. The second is their capacity to incorporate various agents, such as drugs, which further enhance their ability to prevent adhesion formation. Some advantages of planar nanofibrous layers include their potential biodegradability and similarity to the ECM, which aids in supporting tissue regeneration and healing [33]. Wang et al. (2022) developed electrospun nanofibers with super-lubricated nano-skin (SLNM) grown on them. The nanofibers were made of a hexafluoro-isopropanol solution of polylactic acid, which was then electrospun to form the nanofibers. The nanofibers were coated with zwitterionic polymer chains on their surface, known as super-lubricated nano-skin. These nanofibers, with an average fibre diameter of 0.36 μm and water contact angle of almost 0°, exhibit favourable tensile properties, biocompatibility, and friction coefficients below 0.025. Compared to Interceed^®^ and DK-film^®^, the electrospun nanofibers with SLNM were more effective in preventing abdominal adhesions in animal models and had lower production costs. The researchers suggest that the anti-adhesion properties of SLNM arise from its ability to inhibit fibrosis and inflammation. The hydration layer formed by zwitterionic phosphorylcholine groups prevents fibronectin from adhering to SLNM, thereby reducing vinculin expression and preventing fibroblast adhesion. This layer also inhibits the adhesion of inflammatory cells, lowering TNF-α expression and reducing inflammation [50]. Therefore, the major mechanism driving the anti-adhesive properties of SLNM would be its super-lubricated nano-skin component, which reduces fibrin deposition and inflammation.

**Figure 4:**
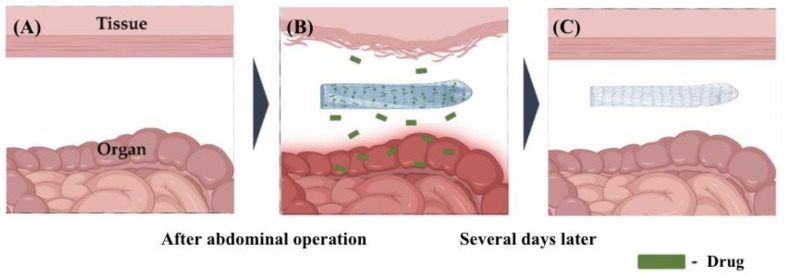
(**A**) This shows the surgical site which involves the tissue and organ surfaces. (**B**) Nanofibers are one of the newer approaches to preventing peritoneal adhesion. Drug-loaded nanofibers further prevent postoperative adhesions through controlled drug release. (**C**) The majority of the drug is released several days following the operation, and the nanofiber is left behind. Adapted from Baek et al. 2020, distributed under the terms and conditions of the Creative Commons Attribution (CC BY) licence (https://creativecommons.org/licenses/by/4.0/) (accessed on 16 December 2024) [52].

Recent studies show that lidocaine has an anti-adhesive effect when loaded onto a poloxamer-alginate-calcium chloride barrier in a rat planar incision model [51]. Building on this, Baek et al. (2020) developed a lidocaine-loaded alginate/carboxymethyl cellulose/PEO nanofiber film. The film was prepared by mixing sodium alginate, sodium carboxymethyl cellulose and PEO in a lidocaine solution. The authors controlled the degree of crosslinking and concentration of calcium chloride (CaCl_2_) to regulate lidocaine release and enhance alginate’s negative charge to reduce cell adhesion. The primary mechanism for reducing adhesion formation is the controlled release of lidocaine, which has been previously established to possess anti-adhesive properties. In addition, alginate’s ability to reduce fibroblast adhesion and, consequently fibrin deposition, supplements the anti-adhesive effects of lidocaine. Lidocaine release from alginate/carboxymethyl cellulose/PEO films crosslinked with 1% CaCl_2_ was observed for more than 7 h. At 1 h, nearly 70% of lidocaine was released, which was higher than films crosslinked with 3% and 5% CaCl_2_. Greater crosslinking slowed the release. Films crosslinked with 5% CaCl_2_ are expected to provide a longer, more stable release. However, as anti-adhesion barriers, these films need extended drug release while staying in place [52]. Figure 4 illustrates the functionality of nanofiber films loaded with drugs.

Dinarvand et al. (2012) fabricated biodegradable nanofibers from poly(caprolactone) (PCL), nonabsorbable poly(ethylsulfone) (PES), poly(lactic-co-glycolic acid) (PLGA), and poly(l-lactide) (PLLA). The authors compared their anti-inflammatory and antiadhesive properties with Interceed^®^. The solutions which were used to produce the PCL and PLLA nanofibers were made by dissolving their polymers in dimethylformamide/chloroform. On the other hand, PLGA and PES were dissolved in dimethylformamide/tetrahydrofuran and dimethylformamide, respectively. All of the membranes exhibited similar diameters and pore sizes, with diameters ranging from 200 nm to 800 nm, and surface pore sizes ranging from 1 μm to 3 μm. The water contact angles for PCL, PES, PLGA, and PLLA are 132° ± 7°, 125° ± 6°, 138° ± 8°, and 135° ± 6°, respectively. PCL and PLGA were particularly noteworthy. PCL was as effective as Interceed^®^ in preventing adhesions with less inflammation, while PLGA had the least inflammation and was the best anti-adhesive agent. Apart from its anti-inflammatory properties, the characteristic that enabled PLGA to be the most effective mechanical barrier in reducing adhesions was its hydrophobic nature, which contributes to its low affinity for cell attachment [53].

Biodegradable PCL, which incorporated an antibiotic, ornidazole, was used to assess its anti-adhesive effect in an animal model. The PCL membrane was prepared by mixing PCL solutions in a mixture of chloroform and dimethylformamide. The study has shown that PCL, with or without ornidazole, decreased tissue adhesion via its role as a mechanical barrier. However, ornidazole significantly reduced adhesions and supported healing. This is due to ornidazole’s ability to eliminate bacteria of gastrointestinal origin from the surgical site, which then prevents the bacteria from promoting the formation of adhesions via inflammation and fibrin deposition. Thus, PCL, which acted as both a mechanical barrier and a vessel for drug delivery, together with the antibiotic, had synergistic effects on the prevention of adhesions. In the study, 0.15 mL of a 25 mg ornidazole drug solution was applied to electrospun nonwoven membranes (2 × 3 cm^2^, ~25 μm thick). Drug release, measured spectrophotometrically, showed about 80% release in 3 h and complete release in 18 h. The rapid initial release likely created a burst effect, helping to prevent infections during early wound healing and supported the success of the barrier system [54].

A recent study incorporated curcumin (CUR) into PCL film casts and electrospun nanofibers, which were prepared using PCL and phospholipids [55]. The CUR-PCL film had a rough surface with no pores or cracks, and a water contact angle of 78.0° ± 0.8°. The CUR-PCL nanofibers showed bead-free, uniform fibres (830 ± 22 nm), a water contact angle of 125.1° ± 1.6°, and porous structures. In vitro drug release over 24 h revealed 9% and 13% release from films and nanofibers, respectively. Nanofibers had slightly higher initial drug release, likely due to their interconnected porosity and larger surface area, allowing for easier diffusion than more compact films [56,57]. By day 30, CUR release was 51% for CUR-PCL films and 45–50% for nanofibers, ensuring controlled drug availability. Both membranes provided sustained release without a burst effect, crucial for suppressing fibroblast proliferation and migration [58]. Sustained CUR release may result from the hydrophobic nature of the PCL [59,60], and CUR [60]. The anti-adhesion effects of various formulations were studied after abdominal surgery in animal models. CUR-loaded films and nanofibers showed better anti-adhesion effects than their blank counterparts, with films outperforming nanofibers. The efficacy of films over nanofibers may be due to their less porous surface and superior mechanical performance. Compared to drug-loaded nanofibers and CUR-PCL film, Seprafilm^®^ showed no significant difference in anti-adhesion [55]. Therefore, the effectiveness of the device can be attributed to two characteristics. Firstly, CUR’s various pharmacological effects, including its ability to reduce inflammation and promote fibrinolysis [61]. Secondly, the nanocharacteristics of the device, such as its porosity and mechanical performance, which enhance its ability to function as an effective barrier.

Jiang et al. (2013) developed a double-layered nanofiber membrane with an inner PCL layer loaded with HA. The HA/PCL inner layer was fabricated by co-electrospinning HA and PCL solutions, while the outer PCL layer was added through sequential electrospinning. The resulting fibres were uniform in size, randomly interconnected, and smooth, without any beads. In rat cecum abrasion models, the double-layered membrane demonstrated significantly better antiadhesive effects than a single-layered PCL membrane. The inner HA/PCL layer efficiently prevented adhesions, likely due to HA’s ability to modulate fibrinolysis. Hyaluronan-based agents are known to reduce adhesions [62], potentially through mechanisms such as physical barrier formation, peritoneal repair stimulation, inflammatory response modulation, and fibrinolysis. However, the exact mechanism remains to be fully elucidated. Nonetheless, the HA incorporated into the inner layer contributes to the device’s anti-adhesive properties, alongside the overall mechanical barrier provided by the double-layered nanofiber. This bi-layer electrospun membrane outperforms the HA-loaded PCL membranes due to its higher rheological behaviour, tensile strength, controlled release, and biocompatibility [63].

Shin et al. (2014) developed PLGA-based nanofibers that release epigallocatechin-3-*O*-gallate (EGCG), aiming to inhibit adhesion formation and accelerate the healing process. These nanofibers were produced by electrospinning PLGA with varying concentrations of EGCG (2%, 4%, and 8% by weight). The average fibre diameter of the EGCG-loaded PLGA membranes ranged from 300 nm to 500 nm, similar to pure PLGA electrospun nanofibers. In an in vivo rat model, the EGCG-loaded PLGA nanofibers showed remarkable efficacy in preventing adhesions, performing comparably to Interceed^®^ and surpassing pure PLGA in effectiveness, particularly for the 8% EGCG formulation. However, there was no significant statistical difference in adhesion formation between the pure PLGA-treated rats and the untreated control group. The success of the device in preventing adhesions is likely attributed to the strong anti-inflammatory and anti-fibrotic effects of EGCG. The 8% EGCG-loaded nanofiber exhibited a minor burst effect of less than 10% on the first day, followed by sustained drug release after 7 days, contributing to its anti-adhesive properties [64].

In another study, researchers blended PLGA with poly(ethylene glycol) (PEG) to reduce PLGA’s high hydrophobicity. The PEG/PLGA nanofibers were prepared by dissolving PLGA in a mixed solution of methamphetamine, acetone, and chloroform, and then adding varying amounts of PEG. The membranes created from this blend effectively prevented fibroblast attachment, proliferation, and the penetration of abdominal structures in a rat cecum model while maintaining biocompatibility and biodegradability. The nanofiber membranes with 5% PEG concentration exhibited the best anti-adhesive properties. These membranes had the narrowest diameter distribution, with an average nanofiber diameter of 0.81 μm, and a water contact angle of 86° ± 1.5°. They also demonstrated the highest tensile strength, a smooth surface, and independent nanofibers with no conglutination. As a result, the 5% PEG-loaded PEG/PLGA nanofibers provided the best physical barrier for preventing adhesions in this study [65], owing to their favourable physical and mechanical characteristics.

Gholami et al. (2021) evaluated the adhesion efficacy of poly(urethane) (PU) nanofibers in a rat cecum abrasion model, assessing the effects macroscopically and histopathologically. The electrospun PU nanofibers had diameters ranging from 200 nm to 1000 nm, with large surface areas, uniform structures, and smooth surfaces. Among the different formulations, the 8% PU nanofibers exhibited the most ideal average fibre diameter of 360 nm, minimal bead formation, and the best adhesion inhibition score. The authors attributed the success of the PU nanofibers in preventing adhesions to several factors. These properties include their high surface area, which enhances interaction with surrounding body fluids, their role as a physical barrier that reduces fibroblast infiltration, and their antibacterial activity, which helps to prevent postoperative inflammation. Furthermore, PU degraded faster than PMS Steripack^®^, a commercially available mesh. This gradual breakdown in the body helps prevent long-term foreign body reactions that could result in adhesions [66]. Table 2 presents a comprehensive overview of the nanotechnological features of the abovementioned nanotherapeutics.

## 4. Regulatory Pathways for Nanotherapeutics

While the field of nanotherapeutics for adhesion prevention is still in its developmental stages, several research studies have been conducted on the topic, although many remain preclinical. These strategies show significant potential and may contribute to a shift toward precision medicine. However, any proposed approach must meet stringent criteria for safety, efficacy, and biocompatibility. Therefore, all potential agents must undergo rigorous evaluation through comprehensive clinical trials before they can be widely implemented for universal use [67]. Furthermore, nanotherapeutics are classified and regulated differently across various jurisdictions, depending on whether they are categorised as investigational new drugs (INDs), medical devices, pharmaceuticals, biologics, or combination products.

In the United States, the development of drugs involves preclinical assessments followed by applications to the United States Food and Drug Administration (FDA) for INDs, new drugs, and abbreviated new drugs [68]. Clinical trials are then conducted to assess the safety, efficacy, and appropriate dosage of the drug [69]. In Europe, the process includes obtaining Clinical Trial Authorization, ensuring compliance with Good Manufacturing Practices (GMP), conducting quality and safety assessments, obtaining an ethics committee approval, and an ongoing monitoring of the trial [70,71,72,73].

Many adhesion barriers are classified as medical devices. In the United States, the FDA regulates these devices, requiring evaluations of safety and efficacy, including biocompatibility and nanotoxicity testing. In Europe, these are governed by Regulation 2017/745, which classifies devices containing nanomaterials as Class III (high risk) unless the nanomaterial is encapsulated to reduce exposure [74].

The FDA recommends Quality by Design for nanopharmaceuticals, while the regulatory pathway for nanobiologics focuses on safety, potency, and characterisation [72]. Combination products are regulated by the FDA with design and risk controls [75].

Some existing nanotherapeutic agents are nearing the clinical trial stage. For example, when preparing the naproxen-loaded chitosan hydrogel, high concentrations of β-GP and acid solvents were used [4]. However, such high concentrations of β-GP may increase the hydrogel’s toxicity. To make the naproxen-loaded chitosan hydrogel safe for future clinical use, it will be necessary to reduce the concentration of β-GP or develop other thermosensitive chitosan hydrogels that do not require acid solubilisation [4].

## 5. Safety and Ethics of Nanotherapeutics

Despite the exciting prospects of using nanotherapeutics, several concerns regarding their safety and associated ethical issues remain, and these must be adequately addressed.

### 5.1. Safety Concerns

Chronic exposure to nanotherapeutics and nanopollution raises concerns that affect various stakeholders, and these issues must be addressed for nanotherapeutics to be widely adopted. Potential adverse effects, including cancers, Parkinson’s disease, cardiovascular issues, Crohn’s disease, and birth abnormalities, have been identified as significant health risks [76]. While the exact mechanisms behind nanomaterial’s accumulation and their link to diseases remain unclear, it is known that a larger surface area increases oxidative stress in nanotherapeutics, heightening the risk of inflammation and cytotoxicity, particularly in the lungs [77,78]. To fully understand the mechanisms underlying these disease process, further detailed studies are required to investigate the effects of chronic exposure to nanomaterials.

Prolonged exposure to nanomaterials without appropriate safety measures may increase risks for manufacturers. As the risks associated with nanomaterials remain unclear, workers must rely on interim precautions for fine and ultrafine particles. Since nanomaterials vary in risk, it is essential to implement ongoing health evaluations, communication, and safety management to ensure worker protection [79].

Furthermore, the manufacturing process of nanomaterials generates waste, contributing to nanopollution [80]. Recent studies indicate that toxic nanomaterials can remain suspended in air and water for days or weeks, potentially posing significant risks during manufacturing, shipping, handling, disposal, and recycling [81]. Moreover, the metal components of nanomaterials in the soil can be transferred to edible plant parts [82], leading to toxic effects on the food chain [83].

Nanoparticle safety is influenced by several factors, including material type, size, and surface charge. For instance, titanium dioxide nanoparticles (TiO-NPs) have been shown to cause cytotoxicity. In contrast, lipid nanoparticles (LNPs) can trigger immunogenicity, inflammation, and toxicity, which are influenced by their composition and surface modifications [84,85]. Smaller nanoparticles possess a higher surface area-to-volume ratio, which increases their reactivity and toxicity. For example, smaller carbon dots (10–20 nm) were found to be more toxic than larger ones (40–100 nm). Similarly, mesoporous silica nanoparticles (mSiNPs) demonstrated size-dependent cytotoxicity [86,87]. Positively charged nanoparticles, such as TiO-NPs and carbon dots, tend to be more toxic [84,86]. Other properties, such as surface hydrophobicity, crystallinity, shape, and agglomeration, also play a significant role in toxicity. For instance, rod-shaped TiO-NPs were more toxic than their spherical counterparts. Surface modifications, such as PEGylation, can improve biocompatibility. Additionally, functionalization of mSiNPs with carboxyl groups has been shown to reduce cytotoxicity compared to pristine particles [84,86,87].

Compared to metal nanoparticles, the nanocomposites, hydrogels and nanofibers, as mentioned earlier, tend to be much safer. For instance, the biosafety of pCNP delivering DXP, silicate nanoplatelets and PEO hydrogels, and SLNM has been confirmed through studies.

### 5.2. Ethical Concerns

The ethical concerns surrounding nanotherapeutics are significant and need careful attention. Two major concerns are unequal access and the potential inability to obtain proper informed consent from patients.

As nanomedicine involves cutting-edge, high-cost technologies, nanotherapeutics are likely to carry a significant cost, which could make them inaccessible to financially disadvantaged patients [88]. Moreover, as developed countries, particularly the United States, European nations, and China, lead the field of nanomedical research [89], they are more likely to secure patents for new nanotherapeutics [88]. This may exacerbate the disparity in access to new medical technologies between the developed and developing worlds. Consequently, patients in developing countries may not be able to benefit from the potential improvements in quality of life that nanotherapeutics could offer.

Furthermore, obtaining true informed consent from participants in nanomedical trials may prove to be challenging. Given the novel and evolving nature of this field, along with the unknown and potentially unpredictable behaviour of nanotechnology in the human body, medical professionals may struggle to fully understand the implications themselves. This could make it difficult for them to provide patients with the thorough explanation needed for informed decision-making [88]. In addition, the risks associated with using nanotherapeutics have not been fully established. This uncertainty increases the likelihood that participants in clinical trials may underestimate the potential risks, further complicating the process of obtaining fully informed consent [90,91].

## 6. Future Perspectives

The use of nanotechnology in preventing postoperative adhesions is an exciting area for future research, offering numerous innovative opportunities. Nanotherapeutics provide many benefits that current therapeutic modalities have yet to cover, including a reduced risk of side effects. However, the biocompatibility of nanotherapeutics requires further investigation to ensure their safety and efficacy. The development of nanotechnology should proceed with careful consideration of ethical and safety concerns to ensure its responsible application. Currently, research on nanotherapeutics in animal models shows great promise, with significant efforts being made to translate these findings into human models for potential clinical use. Further studies will be critical in preventing postoperative adhesions and reducing the healthcare burden associated with their treatment, especially in light of rising global healthcare costs. Therefore, to maximise cost efficiency, which is a universal concern, governments should gain a deeper understanding of the cost-effectiveness of nanotherapeutics [92].

Given the growing interest in biodegradable polymers over non-biodegradable petroleum-based plastics, future nanotherapeutics should prioritise the use of biodegradable polymers such as PLGA. Derived from lactic and glycolic acids, which are sourced from renewable resources, PLGA offers a greener, more sustainable alternative, further aligning with environmental and healthcare sustainability goals [93].

## 7. Conclusions

Postoperative abdominal adhesions develop when the rate of fibrin deposition exceeds the rate of fibrinolysis following surgery. These adhesions can lead to severe complications, including intestinal obstructions and chronic pain, highlighting the need for effective preventive strategies. Current approaches, such as adhesiolysis, pharmaceuticals, inert polymers, and functional biomaterials, have limitations that nanotherapeutics may help to overcome.

Nanotherapeutics offer several advantages, including their potential for precise drug delivery, enhanced biocompatibility, and cost-effective production. Studies in animal models have demonstrated their efficacy in preventing postoperative abdominal adhesions, providing a promising outlook for their clinical application. Although further research and rigorous clinical testing are necessary, these innovative approaches hold significant potential to reduce the long-term healthcare burden associated with postoperative adhesions.

## Figures and Tables

**Table 1:** The clinical trial designs of mechanical barriers, as along with their advantages and disadvantages, have been evaluated. These products are approved by regulatory authorities in Europe and the United States and are commercially available. Recent studies confirm the safety of these products, although their efficacy remains limited.

Product	Type of Product	Trial Design Type	Advantages/Disadvantages	Ref. No.
Seprafilm^®^	Solid, hyaluronate carboxycellulose	Randomised clinical trial which involved 127 patients undergoing uterine myomectomy.	The incidence was significantly reduced in treated patients.	[36]
Interceed^®^	Solid, oxidised cellulose	Randomised multicentre clinical study which involved 63 patients undergoing bilateral pelvic sidewall adhesiolysis.	Reduced the formation of adhesions from 76% to 41%.	[37]
Adept^®^	Liquid, 4% icodextrin	Randomised double blind clinical study which involved 402 patients undergoing laparoscopic gynaecological surgery.	Safe to use and reduces adhesions.	[38]
SprayShield™	Liquid, poly(ethylene glycol)	Randomised prospective multicentre single blind study which involved 11 patients diagnosed with ulcerative colitis or familial adenomatous polyposis.	Reduced incidence and severity of adhesion formation.	[39]
Hyalobarrier^®^	Gel, auto crosslinked hyaluronan gel	Randomised controlled multicentre blinded clinical study consisting of 52 patients undergoing laparoscopic gynaecological surgery.	Safe to use and showed anti-adhesive properties.	[40]

**Table 2:** The nanotechnological features of each nanotherapeutic which allow them to effectively prevent adhesions.

Nanotherapeutic		Nanoparticle Diameter (nm)	Nanofiber Diameter (nm)	Morphology	Pore Size (µm)	Water Contact Angle (°)	Drug Release	Ref. No.
Nanocomposite	PDA-KGF NP with HA	200	-	Globular	-	-	Gradual KGF release.	[44]
	pCNP NP-A	166.1 ± 1.8	-	-	-	-	-	[46]
	pCNP NP-B	175.1 ± 28.7	-	-	-	-	-	
Hydrogel	Col-APG-Cys@HHD	100	-	Spherical	-	-	-	[49]
	NAP-CS	33	-	-	-	-	32% released in 24 h, reaching 68% over 6 days.	[4]
	SNP-PEO	25	-	Disc	-	-	-	[41]
Nanofiber	SLNS	-	360	-	-	0	-	[50]
	LID-loaded SA/CMC/ PEO film	-	-	-	-	-	Films with 1% CaCl_2_: 70% release in 1 h (higher than 3% and 5%).Films with 5% CaCl_2_: expected to ensure longer stable release.	[52]
	PCL	-	549 ± 236	-	1.8 ± 0.8	132 ± 7	-	[53]
	PES	-	621 ± 118		1.5 ± 0.4	125 ± 6		
	PLGA	-	608 ± 170		1.8 ± 0.7	138 ± 8		
	PLLA	-	589 ± 200		2.0 ± 0.9	135 ± 6		
	PCL with ornidazole	-	25,000	-	-	-	80% were released within 3 h, with complete release in 18 h. The rapid initial release likely caused a burst effect.	[54]
	CUR-PCL film	-	-	Rough surface	-	78.0 ± 0.8	9% were released within 24 h, reaching 51% by day 30.	[55]
	CUR-PCL nanofiber	-	830 ± 22	No beads	-	125.1 ± 1.6	13% were released within 24 h, reaching 45–50% by day 30.	
	Bi-layer nanofiber with inner PCL loaded with HA	-	-	Smooth surface with no beads	-	-	Enhanced controlled release.	[63]
	PLGA-EGCG	-	300–500	-	-	-	Minor burst effect of less than 10% on the first day, followed by sustained release after 7 days.	[64]
	PLGA-PEG	-	810	Smooth surface	-	86 ± 1.5	-	[65]
	PU	-	360	Smooth surface with beads	-	-	-	[66]

## Abbreviations

Col-APG-Cys@HHD	collagen aldehydeylated poly(ethylene glycol) cysteine HSA-18His protein and docetaxel
CaCl_2_	calcium chloride
CUR	curcumin
DXP	dexamethasone 21-palmitate
ECM	extracellular matrix
EGCG	epigallocatechin-3-O-gallate
FDA	Food and Drug Administration
HA	hyaluronic acid
IND	investigational new drug
LNPs	lipid nanoparticles
mSiNPs	silica nanoparticles
NDAs	new drug applications
PA	plasminogen activators
PAI	plasminogen activator inhibitors
PAI-1	plasminogen activator inhibitor 1
PCL	poly(caprolactone)
pCNP	photo-crosslinkable nanopatch
PDA-KGF	poly(dopamine) human keratinocyte growth factor
PEG	poly(ethylene glycol)
PEO	poly(ethylene oxide)
PES	poly(ethylsulfone)
PLGA	poly(lactic-co-glycolic acid)
PLLA	poly(l-lactide)
PU	poly(urethane)
SLNM	nanofibers with superlubricated nano-skin
TiO-NPs	titanium dioxide nanoparticles
TNF-α	tumour necrosis factor-alpha
t-PA	tissue plasminogen activator
VEGF	vascular endothelial growth factor
β-GP	beta-glycerolphosphate disodium salt pentahydrate

## References

[1] Park H., Baek S., Kang H., Lee D. (2020). Biomaterials to prevent post-operative adhesion. Materials.

[2] Nahirniak P., Tuma F. Adhesiolysis. StatPearls [Internet]. https://www.ncbi.nlm.nih.gov/books/n/statpearls/article-29/.

[3] ten Broek R.P., Issa Y., van Santbrink E.J., Bouvy N.D., Kruitwagen R.F., Jeekel J., Bakkum E.A., Rovers M.M., van Goor H. (2013). Burden of adhesions in abdominal and pelvic surgery: Systematic review and met-analysis. BMJ.

[4] Wang Y., Pang X., Luo J., Wen Q., Wu Z., Ding Q., Zhao L., Yang L., Wang B., Fu S. (2019). Naproxen nanoparticle-loaded thermosensitive chitosan hydrogel for prevention of postoperative adhesions. ACS Biomater. Sci. Eng..

[5] Foster D.S., Marshall C.D., Gulati G.S., Chinta M.S., Nguyen A., Salhotra A., Jones R.E., Burcham A., Lerbs T., Cui L. (2020). Elucidating the fundamental fibrotic processes driving abdominal adhesion formation. Nat. Commun..

[6] Ellis H., Moran B.J., Thompson J.N., Parker M.C., Wilson M.S., Menzies D., McGuire A., Lower A.M., Hawthorn R.J., O’Brien F. (1999). Adhesion-related hospital readmissions after abdominal and pelvic surgery: A retrospective cohort study. Lancet.

[7] Capella-Monsonís H., Kearns S., Kelly J., Zeugolis D.I. (2019). Battling adhesions: From understanding to prevention. BMC Biomed. Eng..

[8] Hellebrekers B.W., Kooistra T. (2011). Pathogenesis of postoperative adhesion formation. Br. J. Surg..

[9] Wong R.S.Y., Tan T., Pang A.S.R., Srinivasan D.K. (2025). The role of cytokines in wound healing: From mechanistic insights to therapeutic applications. Explor. Immunol..

[10] Furie B., Furie B. (1988). The molecular basis of blood coagulation. Cell.

[11] Levi M., van der Poll T., Büller H.R. (2004). Bidirectional relation between inflammation and coagulation. Circulation.

[12] Esmon C. (2000). The protein C pathway. Crit. Care Med..

[13] Lijnen H.R. (2002). Matrix metalloproteinases and cellular fibrinolytic activity. Biochemistry.

[14] Kisucka J., Butterfield C.E., Duda D.G., Eichenberger S.C., Saffaripour S., Ware J., Ruggeri Z.M., Jain R.K., Folkman J., Wagner D.D. (2006). Platelets and platelet adhesion support angiogenesis while preventing excessive hemorrhage. Proc. Natl. Acad. Sci. USA.

[15] DiPietro L.A. (2016). Angiogenesis and wound repair: When enough is enough. J. Leukoc. Biol..

[16] Saed G.M., Diamond M.P. (2004). Molecular characterization of postoperative adhesions: The adhesion phenotype. J. Am. Assoc. Gynecol. Laparosc..

[17] Imudia A., Kumar S., Saed G., Diamond M. (2008). Pathogenesis of intra-abdominal and pelvic adhesion development. Semin. Reprod. Med..

[18] Koninckx P.R., Gomel V., Ussia A., Adamyan L. (2016). Role of the peritoneal cavity in the prevention of postoperative adhesions, pain, and fatigue. Fertil. Steril..

[19] Stommel M.W., ten Broek R.P., Strik C., Slooter G.D., Verhoef C., Grünhagen D.J., van Duijvendijk P., Bemelmans M.H., den Dulk M., Sietses C. (2018). Multicenter observational study of adhesion formation after open and laparoscopic surgery for colorectal cancer. Ann. Surg..

[20] Krielen P., Stommel M.W., Pargmae P., Bouvy N.D., Bakkum E.A., Ellis H., Parker M.C., Griffiths E.A., van Goor H., ten Broek R.P. (2020). Adhesion-related readmissions after Open and laparoscopic surgery: A retrospective cohort study (scar update). Lancet.

[21] Hellebrekers B.W., Trimbos-Kemper T.C., Trimbos J.B.M., Emeis J.J., Kooistra T. (2000). Use of fibrinolytic agents in the Prevention of Postoperative Adhesion Formation. Fertil. Steril..

[22] Li J., Feng X., Liu B., Yu Y., Sun L., Liu T., Wang Y., Ding J., Chen X. (2017). Polymer materials for prevention of postoperative adhesion. Acta Biomater..

[23] Wei G., Chen X., Wang G., Fan L., Wang K., Li X. (2016). Effect of resveratrol on the prevention of intra-abdominal adhesion formation in a rat model. Cell. Physiol. Biochem..

[24] Bayhan Z., Zeren S., Kocak F.E., Kocak C., Akcılar R., Kargı E., Tiryaki C., Yaylak F., Akcılar A. (2016). Antiadhesive and anti-inflammatory effects of pirfenidone in postoperative intra-abdominal adhesion in an experimental rat model. J. Surg. Res..

[25] Imai A., Takagi H., Matsunami K., Suzuki N. (2010). Non-barrier agents for postoperative adhesion prevention: Clinical and preclinical aspects. Arch. Gynecol. Obstet..

[26] Atta H.M. (2011). Prevention of peritoneal adhesions: A promising role for gene therapy. World J. Gastroenterol..

[27] Reid R.L., Hahn P.M., Spence JE H., Tulandi T., Yuzpe A.A., Wiseman D.M. (1997). A randomized clinical trial of oxidized regenerated cellulose adhesion barrier (Interceed, TC7) alone or in combination with heparin. Fertil. Steril..

[28] Corrales F., Corrales M., Schirmer C.C. (2008). Preventing intraperitoneal adhesions with vitamin E and sodium hyaluronate/carboxymethylcellulose: A comparative study in rats. Acta Cir. Bras..

[29] Ward B.C., Panitch A. (2011). Abdominal adhesions: Current and novel therapies. J. Surg. Res..

[30] Pugliese E., Coentro J.Q., Zeugolis D.I. (2018). Advancements and Challenges in Multidomain Multicargo Delivery Vehicles. Adv. Mater..

[31] Coentro J.Q., Pugliese E., Hanley G., Raghunath M., Zeugolis D.I. (2019). Current and upcoming therapies to modulate skin scarring and fibrosis. Adv. Drug Deliv. Rev..

[32] Cheung J.P., Tsang H.H., Cheung J.J., Yu H.H., Leung G.K., Law W.L. (2009). Adjuvant therapy for the reduction of postoperative intra-abdominal adhesion formation. Asian J. Surg..

[33] Klicova M., Rosendorf J., Erben J., Horakova J. (2023). Antiadhesive nanofibrous materials for medicine: Preventing undesirable tissue adhesions. ACS Omega.

[34] ten Broek R.P., Bakkum E.A., Laarhoven C.J., van Goor H. (2016). Epidemiology and prevention of postsurgical adhesions revisited. Ann. Surg..

[35] Ezhilarasu H., Vishalli D., Dheen S.T., Bay B.-H., Srinivasan D.K. (2020). Nanoparticle-based Therapeutic Approach for diabetic wound healing. Nanomaterials.

[36] Diamond M.P., The Seprafilm Adhesion Study Group (1996). Reduction of adhesions after uterine myomectomy by Seprafilm membrane (Hal-F): A blinded, prospective, randomized, Multicenter Clinical Study. Fertil. Steril..

[37] Sekiba K. (1992). Use of Interceed(TC7) absorbable adhesion barrier to reduce postoperative adhesion reformation in infertility and endometriosis surgery. The Obstetrics and Gynecology Adhesion Prevention Committee. Obstet. Gynecol..

[38] Brown C.B., Luciano A.A., Martin D., Peers E., Scrimgeour A., diZerega G.S., Adept Adhesion Reduction Study Group (2007). Adept (icodextrin 4% solution) reduces adhesions after laparoscopic surgery for adhesiolysis: A double-blind, randomized, controlled study. Fertil. Steril..

[39] Banasiewicz T., Horbacka K., Karoń J., Malinger S., Antos F., Rudzki S., Kala Z., Stojcev Z., Kössi J., Krokowicz P. (2013). Preliminary study with SprayShieldTM adhesion barrier system in the prevention of abdominal adhesions. Videosurg. Other Miniinvasive Tech..

[40] Mais V., Bracco G.L., Litta P., Gargiulo T., Melis G.B. (2006). Reduction of postoperative adhesions with an auto-crosslinked hyaluronan gel in gynaecological laparoscopic surgery: A blinded, controlled, randomized, multicentre study. Human Reprod..

[41] Ruiz-Esparza G.U., Wang X., Zhang X., Jimenez-Vazquez S., Diaz-Gomez L., Lavoie A.-M., Afewerki S., Fuentes-Baldemar A.A., Parra-Saldivar R., Jiang N. (2021). Nanoengineered shear-thinning hydrogel barrier for preventing postoperative abdominal adhesions. Nano-Micro Lett..

[42] Liu H.-J., Wu C.-T., Duan H.-F., Wu B., Lu Z.-Z., Wang L. (2006). Adenoviral-mediated gene expression of hepatocyte growth factor prevents postoperative peritoneal adhesion in a rat model. Surgery.

[43] Kargozar S., Gorgani S., Nazarnezhad S., Wang A.Z. (2023). Biocompatible nanocomposites for postoperative adhesion: A state-of-the-art review. Nanomaterials.

[44] Wei G., Wang Z., Liu R., Zhou C., Li E., Shen T., Wang X., Wu Y., Li X. (2022). A combination of hybrid polydopamine-human keratinocyte growth factor nanoparticles and sodium hyaluronate for the efficient prevention of postoperative abdominal adhesion formation. Acta Biomater..

[45] Lopes J.B., Dallan L.A., Campana-Filho S.P., Lisboa L.A., Gutierrez P.S., Moreira L.F.P., Oliveira S.A., Stolf N.A. (2009). Keratinocyte growth factor: A new mesothelial targeted therapy to reduce postoperative pericardial adhesions. Eur. J. Cardio-Thorac. Surg..

[46] Mi Y., Yang F., Bloomquist C., Xia Y., Sun B., Qi Y., Wagner K., Montgomery S.A., Zhang T., Wang A.Z. (2019). Biologically targeted photo-crosslinkable nanopatch to prevent postsurgical peritoneal adhesion. Adv. Sci..

[47] Ho T.-C., Chang C.-C., Chan H.-P., Chung T.-W., Shu C.-W., Chuang K.-P., Duh T.-H., Yang M.-H., Tyan Y.-C. (2022). Hydrogels: Properties and applications in Biomedicine. Molecules.

[48] Wu S., Hua M., Alsaid Y., Du Y., Ma Y., Zhao Y., Lo C., Wang C., Wu D., Yao B. (2021). Poly(vinyl alcohol) hydrogels with broad-range tunable mechanical properties via the Hofmeister effect. Adv. Mater..

[49] Zhou J., Wang H., Chen H., Ling Y., Xi Z., Lv M., Chen J. (2023). PH-responsive nanocomposite hydrogel for simultaneous prevention of postoperative adhesion and tumor recurrence. Acta Biomater..

[50] Wang Yi Xu Y., Zhai W., Zhang Z., Liu Y., Cheng S., Zhang H. (2022). In-situ growth of robust superlubricated nano-skin on electrospun nanofibers for post-operative adhesion prevention. Nat. Commun..

[51] Choi G.J., Kang H., Hong M.E., Shin H.Y., Baek C.W., Jung Y.H., Lee Y., Kim J.W., Park I.K., Cho W.J. (2017). Effects of a lidocaine-loaded poloxamer/alginate/CACL2 mixture on postoperative pain and adhesion in a rat model of incisional pain. Anesth. Analg..

[52] Baek S., Park H., Park Y., Kang H., Lee D. (2020). Development of a lidocaine-loaded alginate/CMC/PEO electrospun nanofiber film and application as an anti-adhesion barrier. Polymers.

[53] Dinarvand P., Hashemi S.M., Seyedjafari E., Shabani I., Mohammadi-Sangcheshmeh A., Farhadian S., Soleimani M. (2012). Function of poly (lactic-co-glycolic acid) nanofiber in reduction of adhesion bands. J. Surg. Res..

[54] Bölgen N., Vargel Korkusuz P., Menceloğlu Y.Z., Pişkin E. (2006). In vivo performance of antibiotic embedded electrospun PCL membranes for prevention of abdominal adhesions. J. Biomed. Mater. Res. Part B Appl. Biomater..

[55] Babadi D., Rabbani S., Akhlaghi S., Haeri A. (2022). Curcumin polymeric membranes for postoperative peritoneal adhesion: Comparison of nanofiber vs. film and phospholipid-enriched vs. non-enriched formulations. Int. J. Pharm..

[56] Shen X., Xu Q., Xu S., Li J., Zhang N., Zhang L. (2014). Preparation and transdermal diffusion evaluation of the prazosin hydrochloride-loaded electrospun poly(vinyl alcohol) Fiber Mats. J. Nanosci. Nanotechnol..

[57] Tawfik E.A., Scarpa M., Abdelhakim H.E., Bukhary H.A., Craig D.Q., Barker S.A., Orlu M. (2021). A potential alternative orodispersible formulation to prednisolone sodium phosphate orally disintegrating tablets. Pharmaceutics.

[58] Huang Y., Shi R., Gong M., Zhang jingshuang Li W., Song Q., Wu C., Tian W. (2018). Icariin-loaded electrospun PCL/gelatin sub-microfiber mat for preventing epidural adhesions after laminectomy. Int. J. Nanomed..

[59] Hou Z., Li Y., Huang Y., Zhou C., Lin J., Wang Y., Cui F., Zhou S., Jia M., Ye S. (2012). Phytosomes loaded with mitomycin C–soybean phosphatidylcholine complex developed for Drug Delivery. Mol. Pharm..

[60] Ranjbar-Mohammadi M., Bahrami S.H. (2016). Electrospun curcumin loaded poly(ε-caprolactone)/gum tragacanth nanofibers for Biomedical Application. Int. J. Biol. Macromol..

[61] Türkoğlu A., Gül M., Yuksel H.K., Alabalik U., Ülger B.V., Uslukaya O., Avci Y. (2014). Effect of intraperitoneal curcumin instillation on postoperative peritoneal adhesions. Med. Princ. Pract..

[62] Reijnen M.M., Bleichrodt R.P., van Goor H. (2003). Pathophysiology of intra-abdominal adhesion and abscess formation, and the effect of hyaluronan. Br. J. Surg..

[63] Jiang S., Wang W., Yan H., Fan C. (2013). Prevention of intra-abdominal adhesion by Bi-Layer Electrospun Membrane. Int. J. Mol. Sci..

[64] Shin Y.C., Yang W.J., Lee J.H., Oh J.-W., Kim T.W., Park J.-C., Hyon S.-H., Han D.-W. (2014). PLGA nanofiber membranes loaded with epigallocatechin-3-o-gallate are beneficial to prevention of postsurgical adhesions. Int. J. Nanomed..

[65] Li Jian Zhu J., He T., Li W., Zhao Y., Chen Z., Zhang J., Wan H., Li R. (2017). Prevention of intra-abdominal adhesion using electrospun PEG/PLGA nanofibrous membranes. Mater. Sci. Eng. C.

[66] Gholami A., Abdoluosefi H.E., Riazimontazer E., Azarpira N., Behnam M., Emami F., Omidifar N. (2021). Prevention of postsurgical abdominal adhesion using electrospun TPU nanofibers in rat model. BioMed Res. Int..

[67] Fatehi Hassanabad A., Zarzycki A.N., Jeon K., Dundas J.A., Vasanthan V., Deniset J.F., Fedak P.W. (2021). Prevention of post-operative adhesions: A comprehensive review of present and emerging strategies. Biomolecules.

[68] Tyner K.M., Zou P., Yang X., Zhang H., Cruz C.N., Lee S.L. (2015). Product quality for nanomaterials: Current U.S. experience and perspective. WIREs Nanomed. Nanobiotechnol..

[69] Ventola C.L. (2017). Progress in Nanomedicine: Approved and Investigational Nanodrugs. Pharm. Ther..

[70] Souto E.B., Silva G.F., Dias-Ferreira J., Zielinska A., Ventura F., Durazzo A., Lucarini M., Novellino E., Santini A. (2020). Nanopharmaceutics: Part I—Clinical trials legislation and Good Manufacturing Practices (GMP) of Nanotherapeutics in the EU. Pharmaceutics.

[71] Pita R., Ehmann F., Papaluca M. (2016). Nanomedicines in the EU—Regulatory overview. AAPS J..

[72] Csóka I., Ismail R., Jójárt-Laczkovich O., Pallagi E. (2021). Regulatory considerations, challenges and risk-based approach in nanomedicine development. Curr. Med. Chem..

[73] Ehmann F., Sakai-Kato K., Duncan R., Pérez de la Ossa D.H., Pita R., Vidal J.-M., Kohli A., Tothfalusi L., Sanh A., Tinton S. (2013). Next-generation nanomedicines and Nanosimilars: EU Regulators’ initiatives relating to the development and evaluation of nanomedicines. Nanomedicine.

[74] D’Avenio G., Daniele C., Grigioni M. (2024). Nanostructured Medical Devices: Regulatory Perspective and current applications. Materials.

[75] Rocco P., Musazzi U.M., Minghetti P. (2022). Medicinal products meet medical devices: Classification and nomenclature issues arising from their combined use. Drug Discov. Today.

[76] Asmatulu R. (2011). Toxicity of nanomaterials and recent developments in lung disease. Bronchitis.

[77] Nel A., Xia T., Mädler L., Li N. (2006). Toxic potential of materials at the Nanolevel. Science.

[78] Oberdörster G., Oberdörster E., Oberdörster J. (2005). Nanotoxicology: An emerging discipline evolving from studies of Ultrafine Particles. Environ. Health Perspect..

[79] Schulte P.A., Salamanca-Buentello F. (2006). Ethical and scientific issues of nanotechnology in the workplace. Ciência Saúde Coletiva.

[80] Asmatulu R., Zhang B., Asmatulu E., Asmatulu R. (2013). Chapter 3—Safety and Ethics of Nanotechnology. Nanotechnology Safety.

[81] Abaszadeh F., Ashoub M.H., Khajouie G., Amiri M. (2023). Nanotechnology development in surgical applications: Recent trends and developments. Eur. J. Med. Res..

[82] Vittori Antisari L., Carbone S., Bosi S., Gatti A., Dinelli G. (2018). Engineered nanoparticles effects in soil-plant system: Basil (*Ocimum Basilicum* L.) study case. Appl. Soil Ecol..

[83] Fernandes J.P., Mucha A.P., Francisco T., Gomes C.R., Almeida C.M. (2017). Silver nanoparticles uptake by salt marsh plants–implications for phytoremediation processes and effects in Microbial Community Dynamics. Mar. Pollut. Bull..

[84] Kose O., Tomatis M., Leclerc L., Belblidia N.-B., Hochepied J.-F., Turci F., Pourchez J., Forest V. (2020). Impact of the physicochemical features of TiO2 nanoparticles on their in vitro toxicity. Chem. Res. Toxicol..

[85] Yuan Z., Yan R., Fu Z., Wu T., Ren C. (2024). Impact of physicochemical properties on biological effects of lipid nanoparticles: Are they completely safe. Sci. Total Environ..

[86] Fan J., Claudel M., Ronzani C., Arezki Y., Lebeau L., Pons F. (2019). Physicochemical characteristics that affect carbon dot safety: Lessons from a comprehensive study on a Nanoparticle Library. Int. J. Pharm..

[87] Breznan D., Das D.D., MacKinnon-Roy C., Bernatchez S., Sayari A., Hill M., Vincent R., Kumarathasan P. (2018). Physicochemical properties can be key determinants of mesoporous silica nanoparticle potency in vitro. ACS Nano.

[88] Wasti S., Lee I.H., Kim S., Lee J.-H., Kim H. (2023). Ethical and legal challenges in Nanomedical Innovations: A scoping review. Front. Genet..

[89] Bragazzi N.L. (2019). Nanomedicine: Insights from a bibliometrics-based analysis of Emerging Publishing and Research trends. Medicina.

[90] Atalla K., Chaudhary A., Eshaghian-Wilner M.M., Gupta A., Mehta R., Nayak A., Prajogi A., Ravicz K., Shiroma B., Trivedi P. (2016). Chapter 20—Ethical, privacy, and intellectual property issues in nanomedicine. Wireless Computing in Medicine: From Nano to Cloud with Ethical and Legal Implications.

[91] Resnik D.B., Tinkle S.S. (2007). Ethics in Nanomedicine. Nanomedicine.

[92] Weissig V., Guzman-Villanueva D. (2015). Nanopharmaceuticals (part 2): Products in the pipeline. Int. J. Nanomed..

[93] Savin G., Sastourne-Array O., Caillol S., Bethry A., Assor M., David G., Nottelet B. (2024). Evaluation of porous (poly(lactide-co-glycolide)-co-(ε-caprolactone)) polyurethane for use in orthopedic scaffolds. Molecules.

